# Mucus Hypersecretion and Ciliary Impairment in Conducting Airway Contribute to Alveolar Mucus Plugging in Idiopathic Pulmonary Fibrosis

**DOI:** 10.3389/fcell.2021.810842

**Published:** 2022-01-31

**Authors:** Yang Peng, Zhao-Ni Wang, Ai-Ru Xu, Zhang-Fu Fang, Shi-Ying Chen, Xiao-Tao Hou, Zi-Qing Zhou, Hui-Min Lin, Jia-Xing Xie, Xiao Xiao Tang, De-Yun Wang, Nan-Shan Zhong

**Affiliations:** ^1^ State Key Laboratory of Respiratory Disease, National Clinical Research Center for Respiratory Disease, Guangzhou Institute of Respiratory Health, First Affiliated Hospital of Guangzhou Medical University, Guangzhou Medical University, Guangzhou, China; ^2^ Department of Otolaryngology, Yong Loo Lin School of Medicine, National University of Singapore, Singapore, Singapore; ^3^ Guangzhou KingMed Center for Clinical Laboratory Co., Ltd., Guangzhou, China

**Keywords:** idiopathic pulmonary fibrosis, muco-ciliary clearance, MUC5B, conducting airway mucosa, mucus plugs

## Abstract

Idiopathic pulmonary fibrosis (IPF) is a chronic lung disease attributed to the complex interplay of genetic and environmental risks. The muco-ciliary clearance (MCC) system plays a critical role in maintaining the conduit for air to and from the alveoli, but it remains poorly understood whether the MCC abnormalities in conducting airway are involved in IPF pathogenesis. In this study, we obtained the surgically resected bronchi and peripheral lung tissues from 31 IPF patients and 39 control subjects, and we sought to explore the morphologic characteristics of MCC in conducting airway by using immunostaining and scanning and transmission electron microscopy. In the submucosal regions of the bronchi, we found that the areas of mucus glands (MUC5B^+^) were significantly larger in IPF patients as compared with control subjects (*p *< 0.05). In the surface epithelium of three airway regions (bronchi, proximal bronchioles, and distal bronchioles), increased MUC5B and MUC5AC expression of secretory cells, decreased number of ciliated cells, and increased ciliary length were observed in IPF patients than control subjects (all *p *< 0.05). In addition, the mRNA expression levels of MUC5B were up-regulated in both the bronchi and peripheral lung of IPF patients than those of control subjects (*p *< 0.05), accompanied with 93.55% IPF subjects who had obvious MUC5B^+^ mucus plugs in alveolar regions. No MUC5B rs35705950 single-nucleotide polymorphism allele was detected in both IPF patients and control subjects. Our study shows that mucus hypersecretion and ciliary impairment in conducting airway are major causes of mucus plugs in alveolar regions and may be closely related to the alveolar injuries in IPF patients.

## Introduction

### Background

Idiopathic pulmonary fibrosis (IPF) is the most common type of idiopathic interstitial pneumonia characterized by gas exchange impairment, progressive dyspnea, and overall poor prognosis, for which there is restricted treatment options (Mora et al., 2017; Khanna et al., 2020). Although the pathogenesis of IPF remains elusive, strong clinical and experimental evidence indicates that the disease occurs after repetitive alveolar epithelial injuries and failure of alveolar epithelial regeneration, ultimately leading to loss of lung function (Katzen and Beers 2020; Wu et al., 2020). However, the risk factors and molecular mechanisms underlying recurrent alveolar injuries in IPF patients are not fully understood. While there has been a proliferation of interest surrounding the complex interplay of genetic and environmental risks in alveolar injuries and abnormalities of IPF, the role of morphological changes in muco-ciliary clearance (MCC) of conducting airway remains poorly understood (Wiscombe et al., 2013; McDonough et al., 2019).

MCC provides the first line of defense for conducting airway by removing foreign particles through the coordination between secretory cells and ciliated cells (Bustamante-Marin and Ostrowski 2017; Guan et al., 2018). Mucin 5B (MUC5B) and MUC5AC, primarily secreted from secretory cells in submucosal glands or surface epithelium of conducting airway, respectively, are major gel-forming mucins in the healthy airway and play key roles in host defense (Groneberg et al., 2002; Roy et al., 2014). Club cell 10-kDa protein (CC10), a member of the secretoglobin family, is mainly expressed by club cells in the distal bronchiolar epithelium of the peripheral lung (Zuo et al., 2018). Dysregulation of secretory proteins (e.g., MUC5B, MUC5AC, and CC10) and dysfunction of ciliated cells can promote mucus accumulation in the airways, compromising the immune response and perpetuating tissue damage, leading to disease exacerbations (e.g., nasal polyp, asthma, and chronic obstructive pulmonary disease) (Thomas et al., 2010; De Rose et al., 2018; Peng et al., 2019). To date, the highest genetic risk factor for sporadic IPF is a common variant in single-nucleotide polymorphism (SNP) for MUC5B promoter region (rs35705950), which has been confirmed in several independent cohorts, predominantly in Caucasians (Allen et al., 2017; Hobbs et al., 2019). However, it remains elusive whether the MCC abnormalities in conducting airways are common pathologic characteristics in Chinese IPF patients, and further studies to quantify the distribution of secretory cells as well as characterize the morphological features of ciliated cells in conducting airway of these patients are warranted.

In this study, we hypothesized that MCC abnormalities in the conducting airway are common characteristic of IPF patients, which may contribute to mucus plugs in alveolar regions. To this end, we sought to investigate the morphological characteristics of secretory proteins (MUC5B, MUC5AC, and CC10) and ciliated cells in the conducting airway (bronchi, proximal bronchioles, and distal bronchioles) in IPF patients. Furthermore, we also examined the SNP frequency for MUC5B promoter region (rs35705950) and the mRNA expression levels of MUC5B in IPF patients of our Chinese cohort. Our results provided new perspectives on the pathophysiology of IPF and might shed light on the development of novel treatment strategy.

## Methods

### Subject Recruitment and Sample Processing

All subjects were of Han origin from mainland China and provided written informed consent. Study protocol approval was obtained from the institutional review boards of the First Affiliated Hospital of Guangzhou Medical University. Smokers were defined as current or former cigarette smokers if they had smoked for more than 10 pack years. No study participant had a physician’s primary diagnosis of asthma, primary ciliary dyskinesia, or cystic fibrosis.

We recruited 39 control subjects who underwent pulmonary lobectomy due to solitary peripheral carcinoma and 31 end-stage IPF patients who underwent lung transplantation. The diagnostic criteria of usual interstitial pneumonia pattern was determined by high-resolution computed tomography and lung histopathological analysis based on An Official ATS/ERS/JRS/ALAT Clinical Practice Guideline for diagnosis of IPF (Raghu et al., 2018), which excludes other interstitial lung diseases with known causes. Biopsies were taken from the proximal bronchial stumps (primary or lobar bronchi), healthy peripheral lung tissues (>1 cm away from the affected tissue of control subjects), and pathological peripheral lung tissues (fibrotic regions of IPF patients). These biopsies were fixed in paraformaldehyde, glutaraldehyde, or RNAlater™ for further research work.

### DNA Preparation and Single-Nucleotide Polymorphism Genotyping

Genomic DNA of peripheral lung tissues was extracted using QIAamp DNA Blood Mini Kit (QIAGEN Co. Ltd., Germany). We measured the predicted mutated SNP genotype (rs35705950) by using Sanger sequencing. PCR primers for rs35705950 were designed using Primer Premier 5.0 software. The detailed methods have been previously described (Xiong et al., 2016).

### RNA Extraction and Quantitative Real-Time Polymerase Chain Reaction

Total RNA was extracted from frozen bronchi or peripheral lung tissues in RNAlater™ using the RNeasy^®^ Plus Mini Kit (QIAGEN, Hilden, Germany) and reverse transcribed into cDNA using the Maxima Reverse Transcriptase Kit (Thermo Fisher Scientific, Waltham, MA, United States) according to the manufacturer’s protocol. The mRNA expression levels of MUC5B were assessed with quantitative real-time PCR (SYBR Green, YEASEN, Shanghai, China). The relative gene expression was calculated by using the comparative 2^−ΔΔCt^ method, which was normalized against the housekeeping gene (glyceraldehyde 3-phosphate dehydrogenase, GAPDH). Amplification of MUC5B and GAPDH was performed with the following primers: MUC5B forward (5′-ACA​AGT​CCA​TGG​ATA​TCG​TC-3′) and MUC5B reverse (5′-ATT​TGG​TCA​AAC​AGG​ATC​AG-3′); zonula occludens 1 (ZO-1) forward (5′-ACC​AGT​AAG​TCG​TCC​TGA​TCC-3′) and ZO-1 reverse (5′-TCG​GCC​AAA​TCT​TCT​CAC​TCC-3′); E-cadherin forward (5′-TGA​AGG​TGA​CAG​AGC​CTC​TGG​AT-3′) and E-cadherin reverse (5′-TGG​GTG​AAT​TCG​GGC​TTG​TT-3′); and GAPDH forward (5′-ACA​GTT​GCC​ATG​TAG​ACC-3′) and GAPDH reverse (5′-TTT​TTG​GTT​GAG​CAC​AGG-3′).

### Morphometric Analysis

All histologic sections from the control subjects and patients with IPF were digitally scanned by using a digital pathological section scanner (PRECICE500B, Beijing, China). According to a recent paper published by (Okuda et al., 2019), the airways were classified as 1) bronchi [defined as airways with cartilage support and submucosal glands (SMGs)]; 2) proximal bronchioles (defined as airways with no cartilage support or SMGs, surrounded by smooth muscle bands and characterized by an undulating epithelium, with 1–2 mm in diameter); 3) distal bronchioles in control subjects (defined as a structure with a less-wrinkled epithelium compared with the proximal bronchioles, with <1 mm in diameter; and 4) distal bronchioles in IPF patients (defined as a structure with a less-wrinkled epithelium and surrounded by fibrosis in honeycomb cysts, with <1 mm in diameter) (Ikezoe et al., 2021; Stancil et al., 2021).

### Immunofluorescence Staining

Three-micrometer-thick tissue sections were dewaxed in xylene, rehydrated in graded alcohols, and rinsed in distilled water. Sections were then subjected to heat-induced antigen retrieval in Tris–EDTA buffer (pH 9.0) at 95°C for 15 min and cooled at room temperature. Sections were, respectively, incubated with primary polyclonal antibodies of MUC5B (1:100, anti-rabbit, E-AB-15988, Elabscience), MUC5AC (1:100, anti-mouse, MA5-12178, Thermo Fisher Scientific), CC10 (1:500, anti-mouse, sc-365992, Santa Cruz Biotechnology), lysozyme (1:50, anti-mouse, ab36362, Abcam), SPC (1:100, anti-mouse, sc-518029, Santa Cruz Biotechnology), ZO-1 (1:200, anti-mouse, 33–9100, Thermo Fisher Scientific), E-cadherin (1:200, anti-rabbit, #3195, Cell Signaling Technology), and *α*-tubulin (1:500, anti-mouse, ab24610, Abcam), followed by incubation with Alexa Fluor 488-conjugated goat anti-mouse and Alexa Fluor 555-conjugated goat anti-rabbit antibodies (1:500, Life Technologies, Carlsbad, CA, United States) at 37°C for 1 h. The nuclei were visualized by staining with 4′-6-diamidino-2-phenylindole (Life Technologies, Carlsbad, CA, United States). Images were acquired with fluorescence microscopy (Leica DM6, Wetzlar, Germany). The actual fluorescent-positive stained areas of MUC5B, MUC5AC, and CC10 expression in surface epithelium and MUC5B expression in submucosal glands were measured in five randomly selected high-power fields (HPFs) through ImageJ software, respectively.

For negative controls, isotype and concentration matches were substituted for primary antibodies.

### Transmission Electron Microscopy

The biopsy samples of the bronchi and the lung were fixed in a mixed fixative solution containing 2.5% glutaraldehyde and 4% paraformaldehyde, respectively, for 2 h at 4°C, and surface epithelial regions were taken by using ZEISS Axio Zoom V16 stereo light microscope (Carl Zeiss, Brock Michelsen A/S, Denmark), then fixed in 1% osmium tetroxide, dehydrated through a graded series of ethanol (30%, 50%, 70%, 90%, and 100%), and embedded in pure Epon 812 resin. Sections (70 nm) from samples were stained with uranyl acetate and lead citrate. Imaging was performed at 100 kV using a Hitachi JEM-1400 PLUS transmission electron microscope (TEM) (Japan Electron Optics Laboratory Co., Ltd.). Digital images of the specimens were acquired using an EMSIS VELETA G3 CCD camera and analyzed by experienced electron microscopists.

### Scanning Electron Microscopy

Bronchi and lung tissues were fixed in 2.5% glutaraldehyde at 4°C for overnight incubation. Samples were osmicated with 1% osmium tetroxide for 1 h followed by dehydration with gradually increasing ethanol concentrations. Dried samples were mounted onto aluminum stubs, and the surface was sputter-coated with gold for visualization with a FEI Quanta 250 FEG scanning electron microscope (SEM). Representative photomicrographs were taken at various angles so that any error in assessment was minimized because of the tilt of the specimen or other processing artifacts.

### Statistical Analysis

Statistical analyses were conducted with SPSS21.0 software (IBM, Chicago, IL, United States) and GraphPad Prism 6 (GraphPad Software, La Jolla, CA, United States). The Kolmogorov–Smirnov tests and Shapiro–Wilk tests revealed that the data were not normally distributed. The Mann–Whitney two-sided non-parametric test was used as appropriate to compare the continuous variables between two groups. *p* < 0.05 was deemed statistically significant for all analyses.

## Results

### Subject Characteristics

The clinical characteristics of control subjects and IPF patients are shown in Table 1. IPF patients yielded significantly lower percent predicted both of forced expiratory volume in 1 s (mean: 65.20% vs. 103.84%, *p* < 0.001) and forced vital capacity (mean: 70.34% vs. 104.89%, *p* < 0.001) than control subjects. Never-smokers were 45.2% of the IPF patients. The allele frequency for the polymorphism MUC5B rs35705950 was not detected in both control subjects (0/20) and IPF patients (0/20) of our Chinese cohort.

**TABLE 1:** Baseline characteristics of study participants.

	Control subjects	IPF patients
Subjects, *n*	39	31
Sex (M/F), *n*	14/25	27/4
Age, years	47.97 ± 10.73	60.90 ± 9.95
Smokers, *n* (%)	0 (0)	17 (54.8)
BMI (kg/m^2^)^a^	22.19 ± 2.80	21.91 ± 3.34
FVC% predicted^a^	104.89 ± 12.07	70.34 ± 16.52
FEV_1_% predicted^a^	103.84 ± 10.26	65.20 ± 17.45
FEV_1_/FVC (%)^a^	83.72 ± 6.43	80.56 ± 10.44
DLCO% predicted^a^	–	51.42 ± 19.05
Detection methods		
Immunofluorescence, *n*		
Bronchi	23	25
Peripheral lung	39	31
qRT-PCR, *n*		
Bronchi	18	17
Peripheral lung	17	17
SEM		
Bronchi	6	5
Peripheral lung	5	5
TEM		
Bronchi	2	2
Peripheral lung	2	2
Sanger sequencing	20	20

a These information from three IPF patients were missed.

Notes: The data of age, BMI, FVC% predicted, FEV_1_% predicted, FEV_1_/FVC (%), and DLCO% predicted were presented as mean ± standard deviation.

BMI, body mass index; DLCO, diffusing capacity for carbon monoxide; F, female; FEV1, forced expiratory volume in 1 s; FVC, forced vital capacity; M, male; qRT-PCR, quantitative real-time polymerase chain reaction; SEM, scanning electron microscopy; TEM, transmission electron microscopy.

### Hyperplasia of Mucus Glands in the Bronchial Submucosal Region of Idiopathic Pulmonary Fibrosis Patients

**FIGURE 1:**
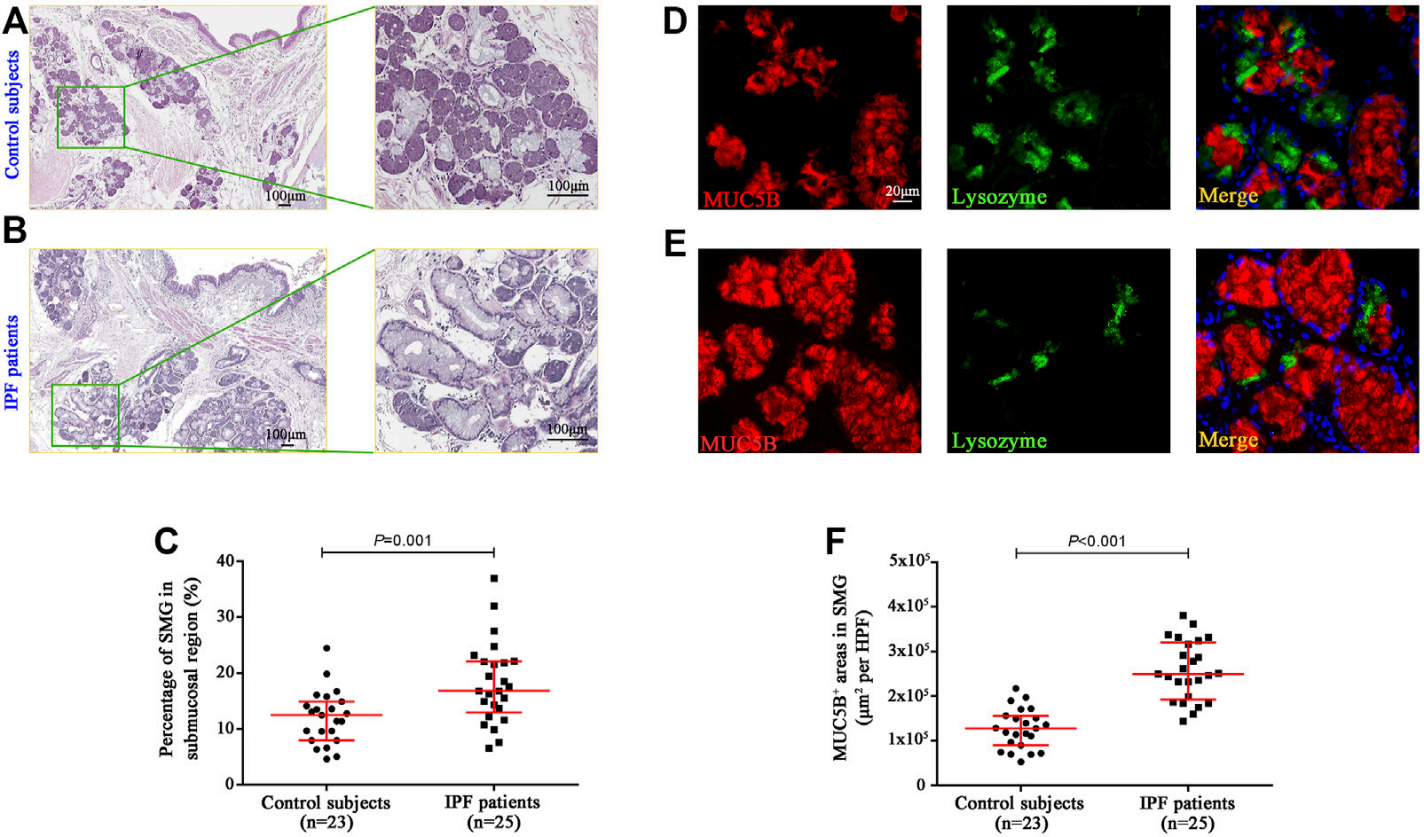
Quantification of glands and MUC5B expression in bronchial submucosal region. Representative H&E staining images in the bronchi from control subjects **(A)** and IPF patients **(B)**, and the median SMG area (expressed as the proportion of SMG region over the total mucosal region) was significantly higher in IPF patients than that in control subjects **(C)**. The IF staining shows that the median areas of mucus component (MUC5B-positive) in SMGs were significantly increased in IPF patients compared to those in control subjects **(D–F)**. DAPI labeling of nuclei (blue). DAPI, 4′,6-diamidino-2-phenylindole; H&E, hematoxylin and eosin; MUC5B, mucin 5B; SMG, submucosal glands; IF, immunofluorescence; IPF, idiopathic pulmonary fibrosis.

To investigate the mucous expression levels in the submucosal region of the bronchi, we respectively assessed the area of SMGs and its mucus component (MUC5B^+^) by using morphometry and image analysis following hematoxylin–eosin staining in five randomly selected HPFs (Figures 1A,B). In control subjects, the median [interquartile range (IQR)] percentage of SMG areas in the bronchial mucosa was 12.45% (7.96%–14.86%), which was significantly lower than 16.81% (12.94%–22.10%) in IPF patients (*p* = 0.001) (Figure 1C). We also found that the median area of MUC5B^+^ mucus glands in SMGs was significantly increased in IPF patients as compared with that in control subjects [24,974.06 μm^2^ per HPF vs. 12,811.17 μm^2^ per HPF (*p *< 0.001)] (Figures 1D,F).

### Overexpression of MUC5B and MUC5AC in Surface Epithelium of the Airways

**FIGURE 2:**
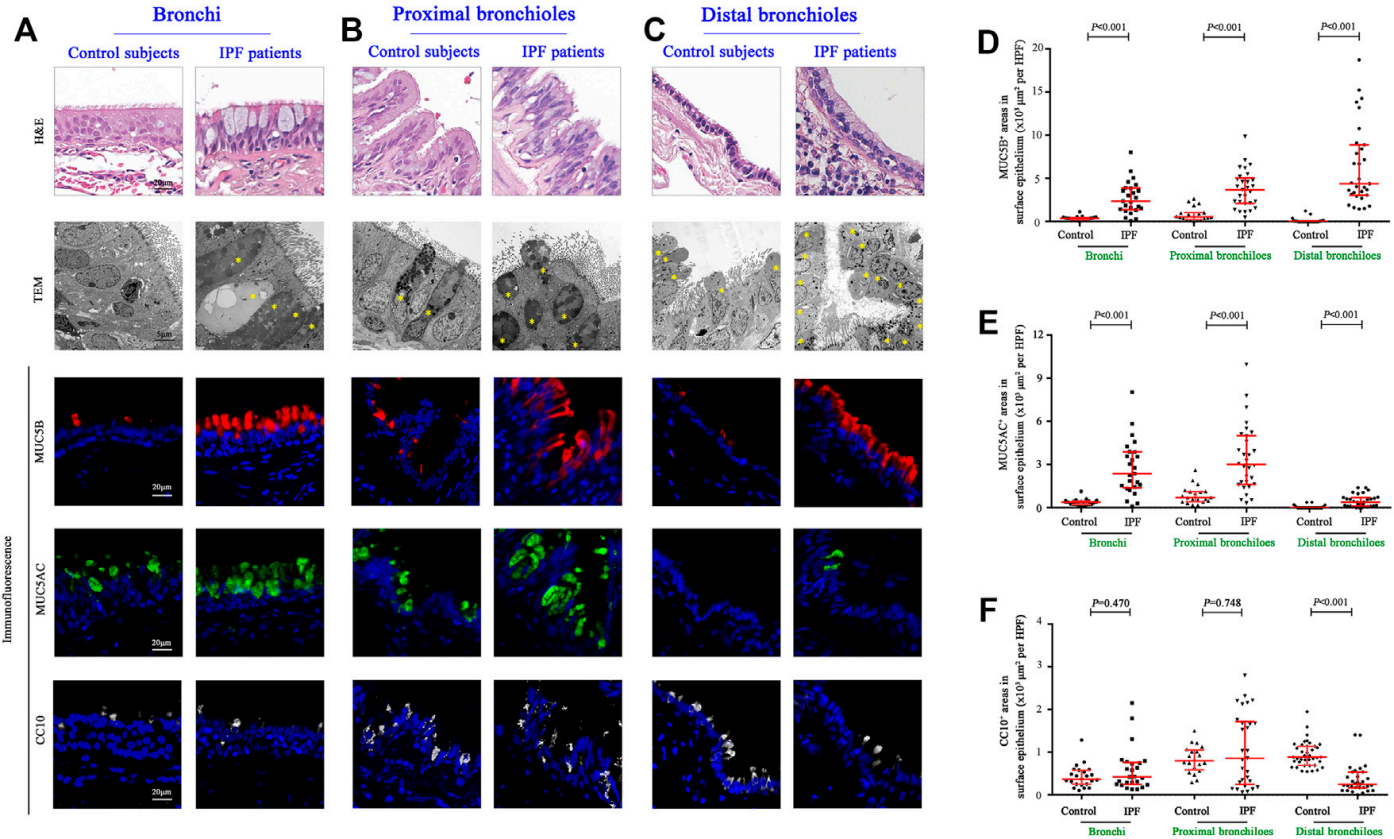
Morphology and expression of secretory proteins in the surface epithelium of the conducting airways. Representative images of H&E staining, TEM, and IF staining showing the morphology and immunopositive secretory cells **(A–C)**; H&E staining and TEM revealed hyperplasia of secretory cells in the epithelium of bronchi and proximal and distal bronchioles from IPF patients, respectively. Quantitation of positive expression areas of MUC5B (red), MUC5AC (green), and CC10 (white) in different airway compartments between control subjects and IPF patients **(D–F)**. DAPI labeling of nuclei (blue). CC10, club cell 10 kDa protein; DAPI, 4′,6-diamidino-2-phenylindole; IF, immunofluorescence; IPF, idiopathic pulmonary fibrosis; MUC5AC, mucin 5AC; MUC5B, mucin 5B; TEM, transmission electron microscope; ns, not significant.

Having revealed a greater proportion in both the area and the mucus component of SMGs in IPF patients, we then explored the stained areas of MUC5B, MUC5AC, and CC10 in the surface epithelium of the bronchi, proximal bronchioles, and distal bronchioles (which was replaced by honeycomb cysts in IPF patients), respectively (Figures 2A–C). In surface epithelium of the three airway compartments, we observed a significantly increased MUC5B and MUC5AC expressions of secretory cells in IPF patients than those of the controls (all *p* < 0.001, Figures 2D,E). Whereas, the expression of CC10^+^ secretory cells was comparable between the two groups in the bronchi and proximal bronchioles, respectively (both *p* > 0.05, Figures 2D,E). Notably, in the surface epithelium of distal bronchioles, we found a significantly lower CC10 expression of secretory cells in IPF patients as compared to that of control subjects (*p* < 0.001, Figure 2F), indicating that the major secretory cell type of CC10^+^MUC5B^−^MUC5AC^−^ in the distal bronchioles of control subjects has been commonly replaced by MUC5B^+^CC10^−^MUC5AC^−^ cells in honeycomb cysts of IPF patients (Figures 2A–C).

### Altered Ciliated Cell Counts and Ciliary Length in Surface Epithelium of Idiopathic Pulmonary Fibrosis Airways

**FIGURE 3:**
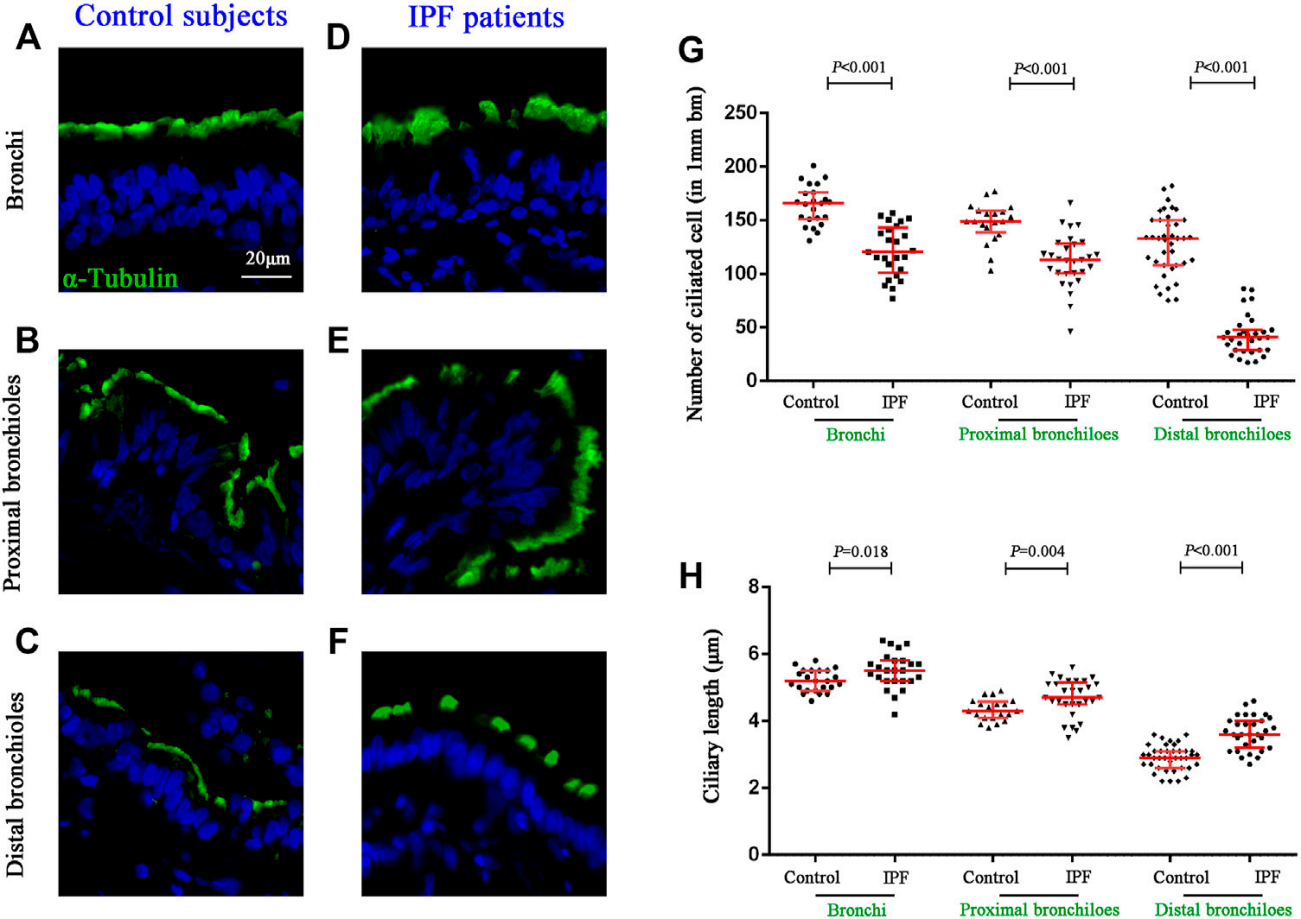
Quantification of ciliated cells and ciliary length in conducting airways. IF staining for *α*-tubulin (stained in green) in different compartments of conducting airway in control subjects **(A–C)** and IPF patients **(D–F)**. The median number of ciliated cells was significantly reduced in all three airway compartments **(G)**, along with the significant increase of ciliary length **(H)** in IPF patients as compared to the controls, respectively. DAPI labeling of nuclei (blue). DAPI, 4′,6-diamidino-2-phenylindole; IF, immunofluorescence; IPF, idiopathic pulmonary fibrosis.

We further sought to determine the distribution of ciliated cells and the length of cilia in the surface epithelium of the conducting airways (Figures 3A–F). Compared with control subjects, the median (IQR) number of ciliated cells per 1 mm basement membrane was significantly reduced in the surface epithelium of the bronchi [165.83 (150.90–175.69) vs. 120.48 (101.11–143.10), *p* < 0.001], proximal bronchioles [149.09 (138.50–158.66) vs. 113.14 (100.42–128.34), *p* < 0.001)], and distal bronchioles [133.42 (108.16–149.96) vs. 40.85 (29.02–47.84), *p* < 0.001] as compared to that in IPF patients, respectively (Figure 3G). Furthermore, the median (IQR) ciliary length (μm) of all airway compartments was significantly increased in IPF patients as compared to that of control subjects {bronchi [5.2 (4.9–5.5) vs. 5.5 (5.2–5.8), *p* = 0.018], proximal bronchioles [4.3 (4.1–4.6) vs. 4.7 (4.4–5.1), *p* = 0.004], and distal bronchioles [2.9 (2.6–3.1) vs. 3.6 (3.2–4.0), *p* < 0.001]} (Figure 3H).

### Mucus Plugs in the Alveolar Regions of Idiopathic Pulmonary Fibrosis Patients

**FIGURE 4:**
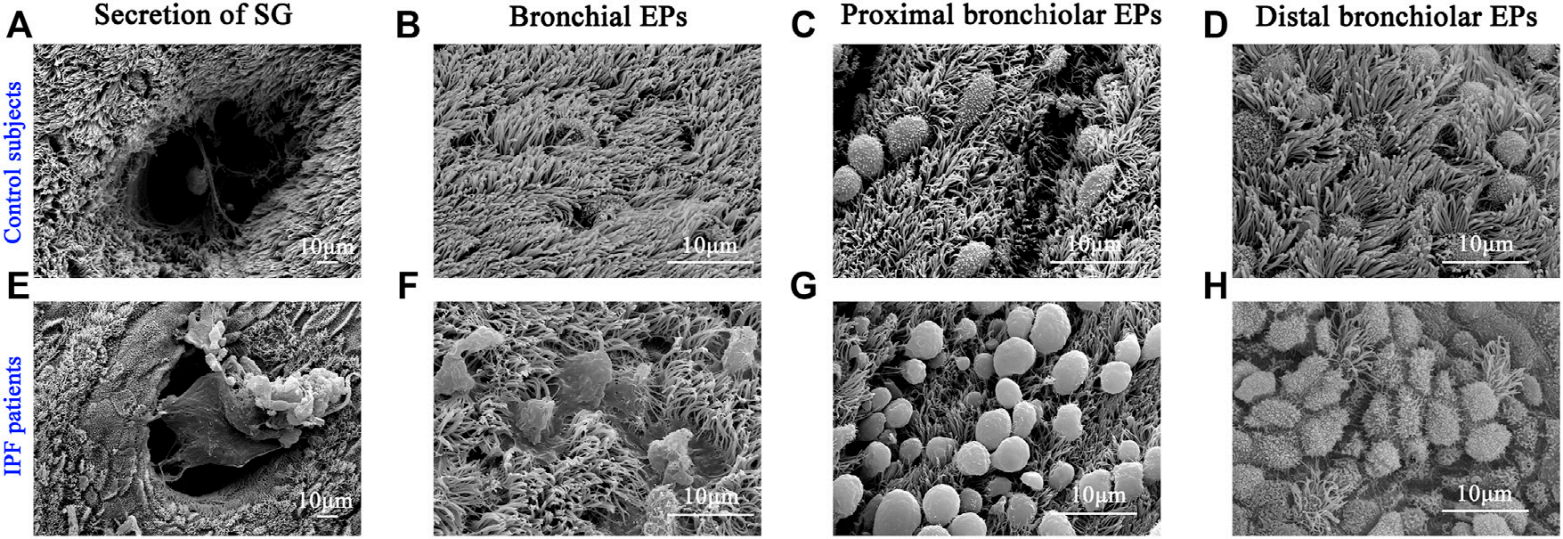
Characteristics of surface epithelium revealed by SEM. SEM was performed to show the distribution of ciliated cells and secretory cells in different regions of conducting airway from control subjects **(A–D)** and IPF patients **(E–H)**. IPF, idiopathic pulmonary fibrosis; SEM, scanning electron microscope. EPs, epitheliums; SG, submucosal gland.

**FIGURE 5:**
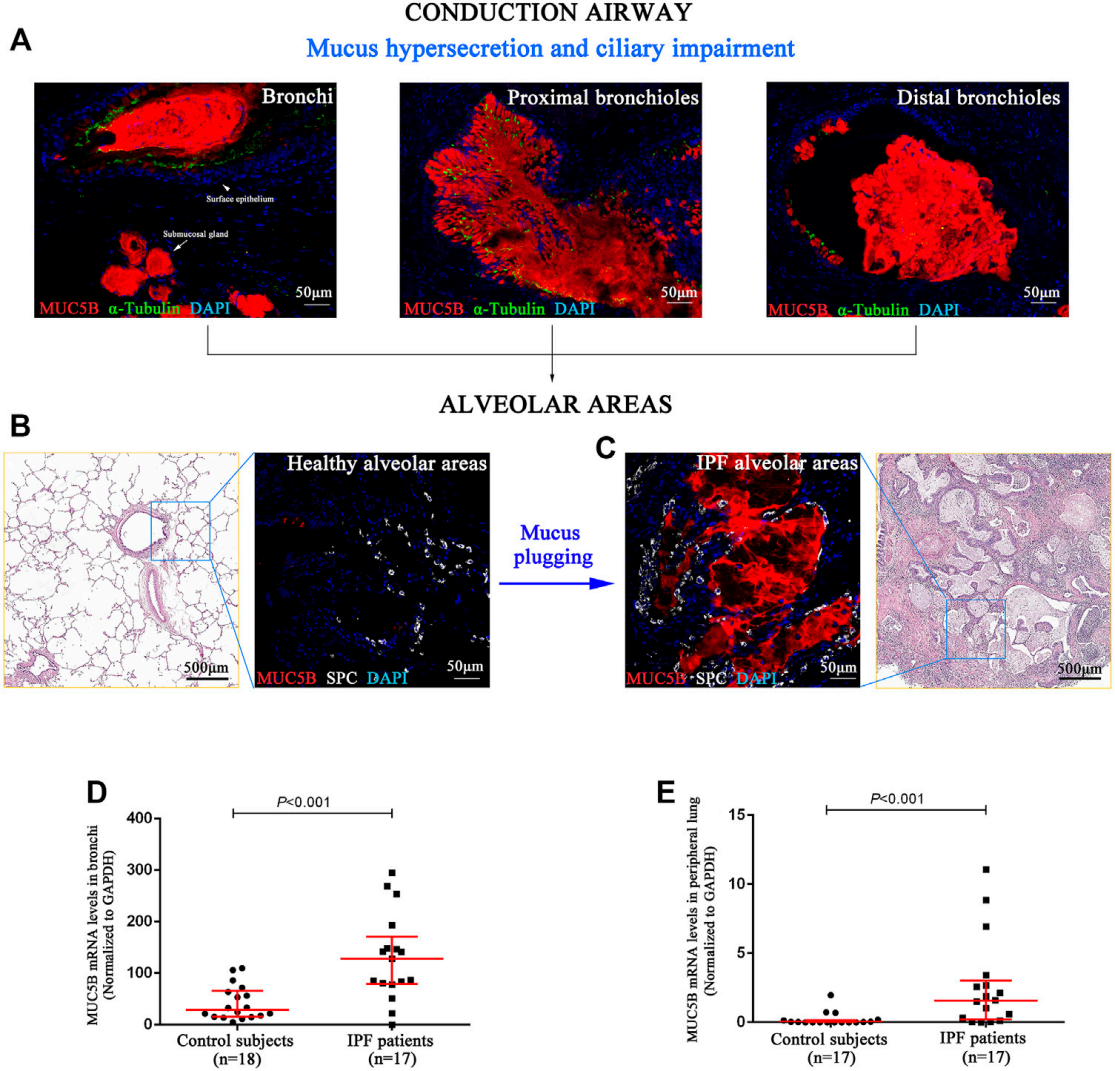
MUC5B^+^ mucus plugging in conducting airway lumens and alveolar regions. The IF staining showing obvious MUC5B^+^ mucus (red) secreted in conducting airway lumens in IPF patients **(A)**. A representative H&E staining of peripheral lung tissue from a control subject, showing that both the distal bronchioles and alveoli have normal size and architecture, without fibrosis or inflammation around **(B)**. A representative H&E staining of honeycomb fibrosis (characterized by cystic fibrotic airspaces that are frequently lined by bronchiolar epithelium and filled with mucus and inflammatory cells) in peripheral lung tissue from an IPF patient **(C)**. Compared with control subjects **(B)**, we found obvious MUC5B^+^ mucus plugging in alveolar regions (SPC-positive for alveolar type 2 cell) **(C)**. As compared to the controls, the MUC5B mRNA levels were significantly higher in both the bronchi **(D)** and peripheral lung **(E)** tissues of IPF patients, respectively. DAPI labeling of nuclei (blue). DAPI, 4′,6-diamidino-2-phenylindole; IF, immunofluorescence; IPF, idiopathic pulmonary fibrosis; MUC5B, mucin 5B; SPC, surfactant protein C.

Next, we performed SEM to reveal the aberrant characteristics of surface epithelium in conducting airway from IPF patients. Our results confirmed the excessive mucus production in SMGs, hyperplasia of secretory cells, and reduced number of ciliated cells in the surface epithelium of conducting airway (Figures 4A–H). By using IF staining, we further demonstrated over-secretion of MUC5B^+^ mucus in the conducting airway lumens of IPF patients (Figures 5A–C), accompanied with 93.55% (29/31) IPF patients that had obvious MUC5B^+^ mucus plugs in alveolar regions surrounded by fibrosis (Figure 5C).

In addition, we performed quantitative real-time PCR on bronchi and peripheral lung tissues derived from control subjects and IPF patients and found that the MUC5B mRNA level was markedly higher in both the bronchi and peripheral lung of IPF patients than that of the control subjects (both *p* < 0.001) (Figures 5D,E).

## Discussion

IPF, a progressive disease that mainly occurs in older adults, resulted in significant morbidity and mortality due to hypoxemic respiratory insufficiency (Fingerlin et al., 2013). The pathogenesis of IPF remains a field of active investigation, and it is generally accepted that disease pathobiology results from repetitive injuries to the alveolar epithelium (Parimon et al., 2020). In the progression phase, the normal alveolar structure is lost and replaced by remodeled fibrotic regions (Wolters et al., 2018). Injury to alveolar epithelia is now generally regarded as a consequence of multiple interactions among ageing, genetic susceptibility, and environmental risk factors (e.g., passive smoking and accumulation of air particulate matter) (Sgalla et al., 2018; Yanagihara et al., 2019). So far, the treatment of IPF remains challenging, and numerous drugs have been tested in multiple clinical trials without success (Selman and Pardo 2020). A detailed investigation of the relationship between IPF and defective host defense responses of conducting airway may help to reveal new targets for therapeutic interventions (Bingle 2011). Our study provided novel insights into the disease pathogenesis that mucus hypersecretion and ciliary impairment are common characteristics in conducting airway of IPF patients and are the major cause of mucus plugging in peripheral lung. Mucus plugging may further lead to repeated micro-injuries and chronic inflammation in alveolar regions, resulting in scar tissue formation, persistent fibroproliferation, and, hence, development of IPF (Hancock et al., 2018; Kurche et al., 2019).

The airway epithelium plays a critical role in maintaining the conduit for air to and from the alveoli. One potential consequence of aberrant MCC in conducting airway is retention of inhaled substances (micro-organisms, cigarette smoke, air pollutants, etc.), leading to chronic inflammation in alveolar regions and worse health-related quality of life (Yang et al., 2021). Alternatively, overexpression of mucus or hyperplasia of mucus-producing cells in the conducting airway may also promote the mucous aspirated into the distal airway and thus leading to gas exchange impairment (Leitz et al., 2021). Aspirated mucous injures alveolar epithelium either through interfering the interaction of type II alveolar epithelial cells and the underlying matrix or by impairing the surface-tension properties of the surfactant (Zhang et al., 2019). These alveolar injuries could result in the collapse and fibrosis of bronchoalveolar units and eventually lead to the development of IPF (Naikawadi et al., 2016; Wu et al., 2020).

Previous studies reported that MUC5B and MUC5AC were secreted at high levels in cigarette smoking, asthmatic inflammation, as well as chronic obstructive pulmonary disease, and suppression of the production of these proteins may be a therapeutic strategy for secretory chronic airway inflammatory diseases (Kesimer et al., 2017; Kim et al., 2019; Ramsey et al., 2020). To determine whether smoking status contributes to the mucus hypersecretion and cilia impairment that we observed in the conducting airway of IPF patients, we separated IPF patients to smokers/non-smokers and performed an additional analysis (Supplementary Figure S1). We found that MUC5AC mRNA expression was increased in the proximal bronchioles of IPF smokers as compared to IPF non-smokers, whereas no alteration was observed in the bronchi or distal bronchiole epithelium. There was also no significant difference in MUC5B and CC10 expression, ciliated cell number, and motile cilia length between IPF smokers and non-smokers. Thus, smoking may only have a slight impact on the mucociliary abnormality in the end-stage IPF lungs. Perl et al. (2011) provided evidence that repeated injuries to bronchiolar CC10^+^ club cells are sufficient to cause pulmonary fibrosis at sites of insufficient repair and reepithelialization. In our study, several lines of evidence link mucins to IPF disease, suggesting that they are also key effectors of the disease. To our knowledge, this is the first study that systematically investigated the expression patterns of MUC5B, MUC5AC, and CC10 in conducting airway of Chinese IPF patients. As shown in Supplementary Figure S2, hyperplasia of MUC5B^+^MUC5AC^+^CC10^−^ cells were observed in IPF patients as compared to the controls, both at the bronchi and proximal bronchioles (Supplementary Figures S1A,B). Furthermore, we found decreased numbers of MUC5B^−^MUC5AC^−^CC10^+^ cells, hyperplasia of MUC5B^+^MUC5AC^−^CC10^−^ cells, and MUC5B^+^MUC5AC^+^CC10^−^ cells in distal bronchioles in patients with IPF (Supplementary Figure S1C). However, the mechanisms mediating intracellular signaling of mucins in IPF development remain unknown. By using a genome-wide linkage scan, a common polymorphism in the promoter variant rs35705950 of MUC5B is found to be associated with familial interstitial pneumonia and IPF in non-Hispanic white patients (Seibold et al., 2011), and it further confirmed that variant rs35705950 affects MUC5B expression in the distal airways of the IPF lung (Nakano et al., 2016). Conversely, since variant rs35705950 was found to be rare in Asian cohort of IPF patients (Horimasu et al., 2015; Peljto et al., 2015; Deng et al., 2018), the excessive production of MUC5B^+^ mucus was unlikely to be associated with this promoter mutation in our Chinese cohort. Therefore, it will be important for future studies to confirm the mechanism of mucus hypersecretion in the conducting airway of Chinese IPF patients and to determine whether these can be manipulated to therapeutic advantage.

Many cellular processes are dependent on the proper timing of ciliogenesis or on the proper ciliary maintenance, and impaired ciliated cells are potential causes of chronic mucosal inflammation or infection in chronic airway diseases (e.g., rhinosinusitis and asthma) (Peng et al., 2018). To our knowledge, this is also the first quantitative study reporting the different characteristics of ciliated cells between the proximal bronchioles and distal bronchioles and indicated significantly reduced number and increased length of ciliated cell in conducting airway of IPF patients. Yang et al. (2013) reported that the expression of cilium genes appears to not only identify two unique molecular phenotypes of IPF but also provide further support for the importance of cilia in this disease. In addition, our study also examined the epithelial junctions, including adherens junction (E-cadherin) and tight junction (ZO-1), which may be closely related to the impairment of MCC in IPF patients. As shown in Supplementary Figure S3, we found that the mRNA levels of E-cadherin were significantly higher in both bronchi (A) and lung tissues (B) of IPF patients as compared to controls. For the mRNA level of ZO-1, no remarkable difference was found in the bronchi between controls and IPF patients (C) but was significantly higher in lung tissues of IPF patients as compared to controls (D). By immunofluorescent staining, we found an untidy distribution and decreased expression of both E-cadherin and ZO-1 in surface epithelium of the conducting airway (bronchi, proximal bronchioles, and distal bronchioles) from IPF patients (E–F). Further studies to understand the detailed molecular mechanisms of ciliary impairment and epithelial junctions in IPF patients are warranted.

Whether the mucociliary abnormalities are the driving force or the consequence of IPF is still undetermined, as we can hardly collect lung samples with early change from IPF patients and perform study. The gain-of-function variant of MUC5B promoter is an acknowledged risk factor for the development of IPF (Seibold et al., 2011), and one animal study also evidenced that selective overexpression of MUC5B in bronchoalveolar epithelial cells augmented bleomycin-induced lung fibrosis (Hancock, et al., 2018). Recently, Stancil et al. (2021) have demonstrated that one copy (GT) of MUC5B promoter variant prolonged the persistence of amphiregulin-induced unjammed phase in IPF-derived airway epithelial cells as compared to the no copy (GG) variant, while epithelial cells with this unjammed phase promoted proliferation and activation of human lung fibroblasts. As for the ciliary abnormality, it is unclear whether it happened before or after the disease onset. The expression of cilium genes was closely correlated to MUC5B level and the extent of honeycombing in IPF patients, such as DNAH6 and DNAH7, which was associated with the cilia motility (Yang et al., 2013), and the biological module of cilia genes can also predict the survival of IPF patients (McDonough et al., 2019). Currently, there is scarce of evidence suggesting the relationship between motile cilia impairment and the development of pulmonary fibrosis, while several factors dysregulated in IPF have been reported to interfere ciliogenesis or ciliated cell differentiation, such as MMP7 (Yang et al., 2013; Gharib et al., 2013).

Our study has some limitations. First, the control subjects were recruited from patients who underwent resection for solitary peripheral carcinoma. Ideally, sampling the tissues from the donors of lung transplantation as the control would be preferred. Second, all the lungs came from patients with end-stage IPF, and the relationship between aberrant MCC and progression of the disease could not be observed over time. Finally, further *in vivo* and *in vitro* investigation is also merited to determine ciliary beating frequency in IPF patients and to explore whether restored MCC in conducting airway could reverse alveolar injuries and disease progression.

In summary, we revealed that the mucus hypersecretion and ciliary impairment in conducting airway are common characteristic of IPF patients, which may be closely related to progressive alveolar injuries. Targeting secretory cells and ciliated cells might represent a promising strategy in the management of IPF, which warrants further investigations. We believe that novel preventive interventions, evolving the use of screening biomarkers, and targeting newly discovered risk factors for IPF would eventually lead to a decline in the incidence, improve the quality of life, and prolong the life-span of IPF patients.

## Data Availability

The original contributions presented in the study are included in the article/Supplementary Material, further inquiries can be directed to the corresponding authors.

## Glossary

BMI: body mass index
COPD: chronic obstructive pulmonary disease
CC10: club cell 10-kDa protein
DAPI: 4′,6-diamidino-2-phenylindole
DLCO: diffusing capacity for carbon monoxide
**FEV**_**1**_: forced expiratory volume in 1 s
FVC: forced vital capacity
GAPDH: glyceraldehyde 3-phosphate dehydrogenase
HPFs: high-power fields
IPF: idiopathic pulmonary fibrosis
IF: immunofluorescence
MCC: muco-ciliary clearance
MUC5AC: mucin 5AC
MUC5B: mucin 5B
qRT-PCR: quantitative real-time polymerase chain reaction
SEM: scanning electron microscopy
SNP: single-nucleotide polymorphism
SPC: surfactant protein C
TEM: transmission electron microscopy
ZO-1: zonula occludens 1
EPs: epitheliums
SG: submucosal gland
