# Supplementary figures and images for: Mucus Hypersecretion and Ciliary Impairment in Conducting Airway Contribute to Alveolar Mucus Plugging in Idiopathic Pulmonary Fibrosis
Source: Front Cell Dev Biol. 2022 Jan 31;9:810842. doi: 10.3389/fcell.2021.810842 (PMC8842394; doi:10.3389/fcell.2021.810842)

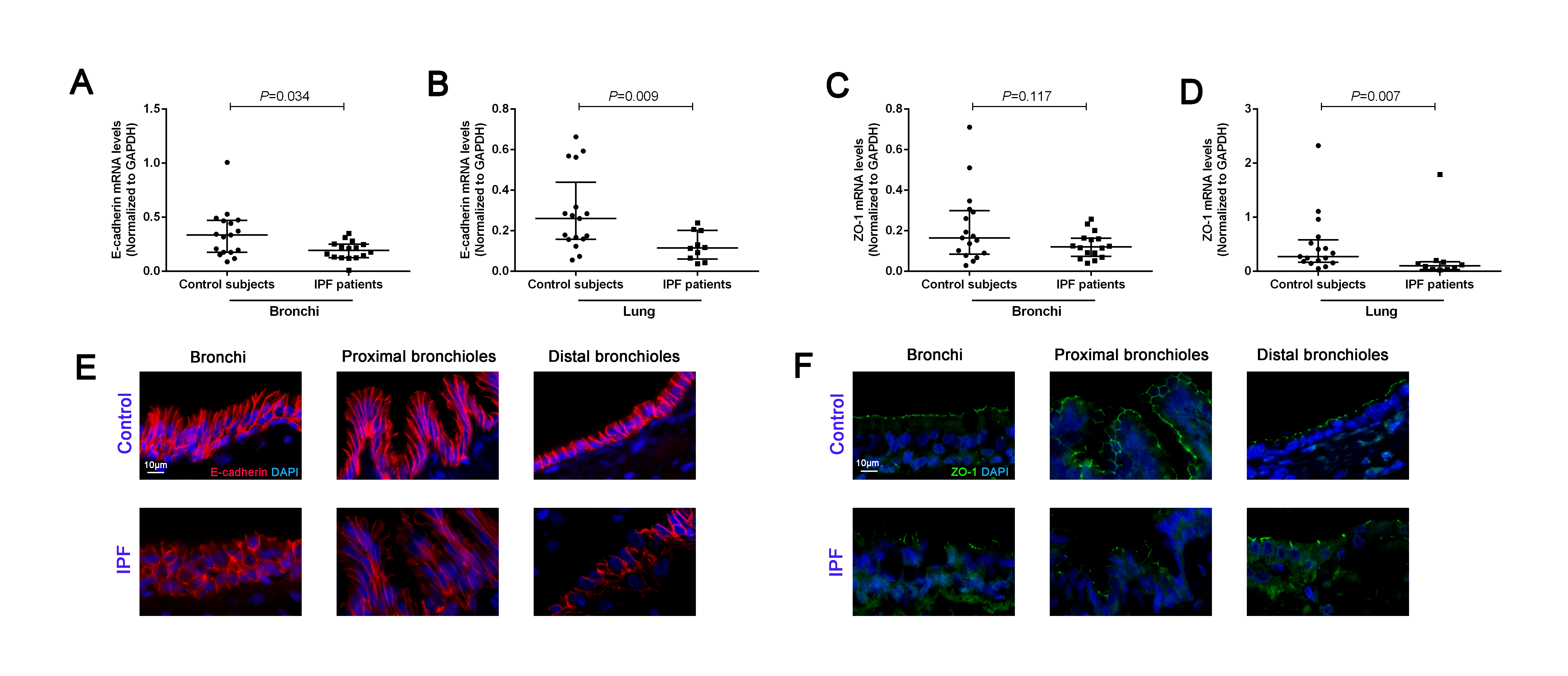

Supplement: Supplementary file 1 [file Image3.JPEG]

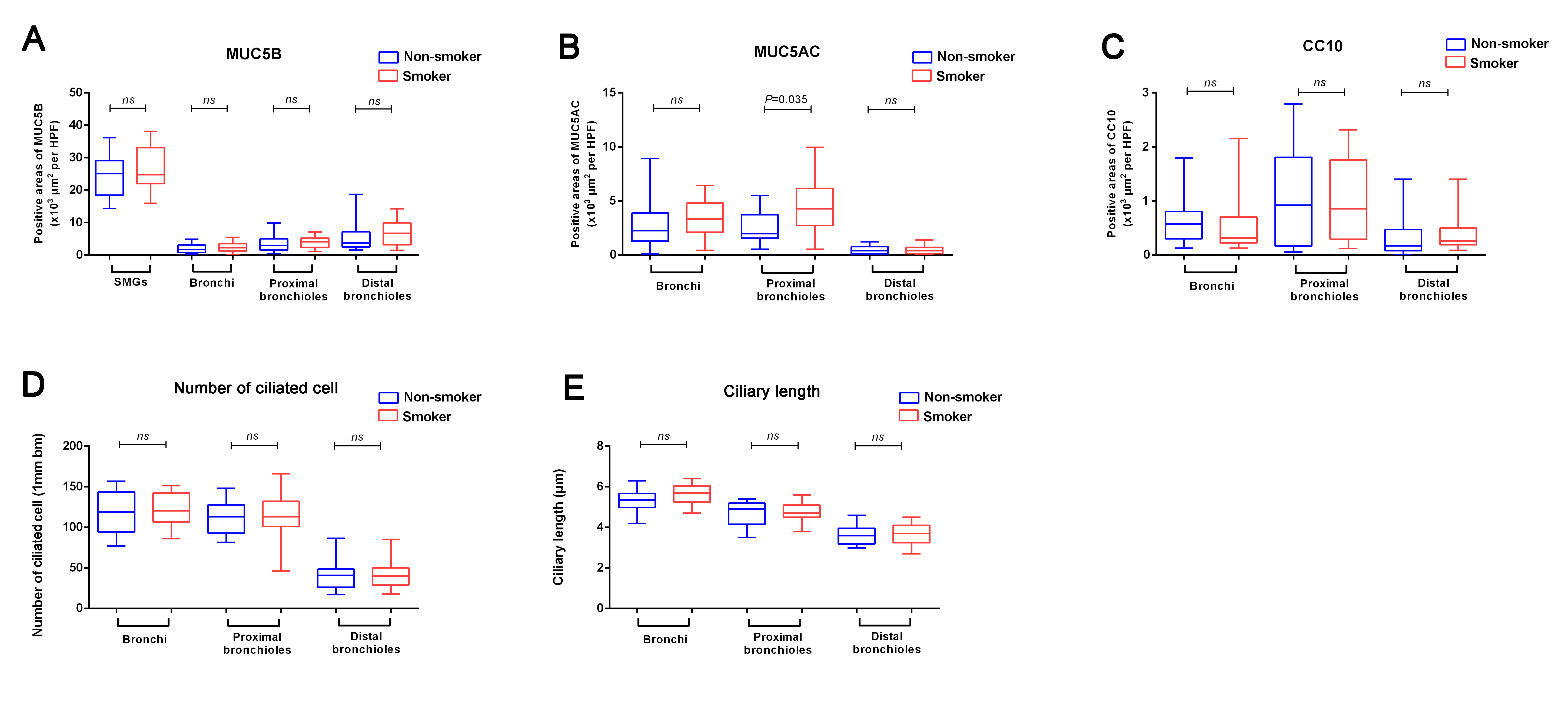

Supplement: Supplementary file 2 [file Image1.JPEG]

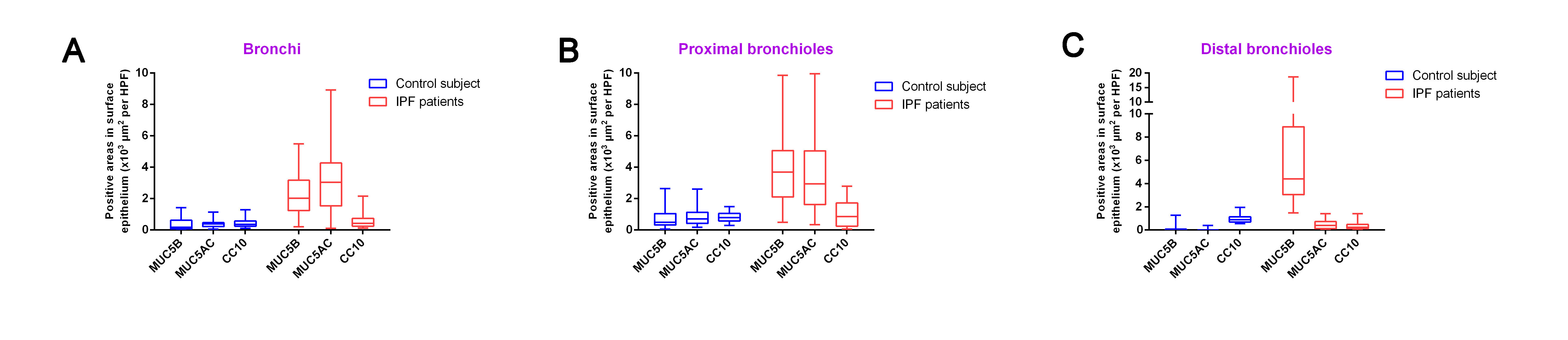

Supplement: Supplementary file 3 [file Image2.JPEG]
