# Supplementary material for: Mucus Hypersecretion and Ciliary Impairment in Conducting Airway Contribute to Alveolar Mucus Plugging in Idiopathic Pulmonary Fibrosis
Source: Front Cell Dev Biol. 2022 Jan 31;9:810842. doi: 10.3389/fcell.2021.810842 (PMC8842394; doi:10.3389/fcell.2021.810842)
Supplement: Supplementary file 4 [file DataSheet10.DOCX]

**SUPPLEMENTAL FIGURE LEGENDS**

**Figure S1. Association between the smoking status and mucus hypersecretion or cilia impairment in conducting airway of IPF patients.**

(A) No significant difference of MUC5B positive expression areas in conducting airway (including SMGs, bronchi, proximal/distal bronchioles) between IPF smokers (n=17) and non-smokers (n=14).

(B) MUC5AC positive expression areas have increased significantly in the proximal bronchioles of IPF smokers (n=17) as compared to IPF non-smokers (n=14), whereas no alteration of MUC5AC mRNA expression level was observed in bronchi or distal bronchiole epithelium.

(C-E) No significant difference of positive expression areas, number of ciliated cells and ciliary length was found in conducting airway (including SMGs, bronchi, proximal/distal bronchioles) between IPF smokers (n=17) and non-smokers (n=14).

CC10: club cell 10kDa protein; HPF: high-power fields; IPF: idiopathic pulmonary fibrosis; MUC5AC: mucin 5AC; MUC5B: mucin 5B; ns, not significant.

**Figure S2. The alteration of secretory cell types in surface epithelium of conducting airways in patients with IPF**

Hyperplasia of MUC5B^+^MUC5AC^+^CC10^-^ cells were observed in IPF patients as compared to the controls, in both the bronchi and proximal bronchioles (A-B). Furthermore, we found decreased numbers of MUC5B^-^MUC5AC^-^CC10^+^ cells, hyperplasia of MUC5B+MUC5AC-CC10- cells, and MUC5B^+^MUC5AC^+^CC10^-^ cells in distal bronchioles in patients with IPF (C).

CC10: club cell 10kDa protein; IPF: idiopathic pulmonary fibrosis; MUC5AC: mucin 5AC; MUC5B: mucin 5B.

**Figure S3. Decreased expression levels of both mRNA and protein of E-cadherin and ZO-1 in IPF patients**

The mRNA expression levels of E-cadherin were significantly higher in both bronchi (A) and lung tissues (B) of IPF patients (n=16 in bronchi and n=10 in lung tissues) as compared to controls (n=17 in bronchi and n=17 in lung tissues). For the mRNA expression levels of ZO-1, no significant difference was found in bronchi between controls and IPF patients (C), but significantly higher in lung tissues of IPF patients as compared to controls (D). By using immunofluorescent staining, we found both of E-cadherin and ZO-1 expression levels are untidy and decreased in surface epithelium of the conducting airway (bronchi, proximal bronchioles and distal bronchioles) from IPF patients (E-F).

DAPI: 4′,6-diamidino-2-phenylindole; IPF: idiopathic pulmonary fibrosis; ZO-1: zonula occludens 1.
